# Exploring Challenges Related to Breast Cancer to Identify Opportunities for Advocacy in Hawassa City, Southern Ethiopia: A Community-Based, Qualitative Study

**DOI:** 10.1200/GO.23.00137

**Published:** 2023-11-16

**Authors:** Netsanet Bogale, Bargude Balta, Gulema Demissie, Dereje Geleta, Michele Rakoff, Brandon Anderson, Natalie Johnson, Lisa Yee, Lesley Taylor

**Affiliations:** ^1^ Hawassa University Comprehensive Specialized Hospital-Cancer Treatment Center, Hawassa, Ethiopia; ^2^ Department of Public Health, Hawassa University College of Medicine and Health Sciences, Hawassa, Ethiopia; ^3^ Breast Cancer Care and Research Fund, Los Angeles, CA; ^4^ Ironwood Cancer and Research Centers, Mesa, AZ; ^5^ City of Hope Comprehensive Cancer Center, Duarte, CA

## Abstract

**PURPOSE:**

The aim of this study was to explore breast cancer (BC) challenges to identify opportunities for advocacy in southern Ethiopia in 2022.

**METHODS:**

Twenty-five participants from four local districts (kebeles) in Hawassa City were selected as key contributors to future work. Semistructured in-depth interviews were held for two clinicians, two local health bureau managers, two media managers, and three religious leaders. Two focus group discussions were conducted: one included six BC survivors and a caregiver; the other included two health extension workers, three members of the Women's Development Group, two community volunteers, one kebele leader, and one traditional healer.

**RESULTS:**

To our knowledge, our study was the first time that most participants had assembled. Many referred to patients as victims and BC as a killer disease or curse. Community and religious leaders were concerned about challenges and willing to collaborate. Survivors, providers, and religious leaders were identified as key sources of information, positive messages, and leadership.

**CONCLUSION:**

Recommendations for advocacy work in Hawassa include lobbying for BC as a health priority; including BC within the health extension package; initiating programs for earlier detection; educating the community to remove stigmas of the disease and treatments; working with media to disseminate messages that are inclusive of people in remote areas and speaking different languages; improving availability, affordability, and access to care; and assisting patients with psychosocial support. A strategic collaboration between religious leaders and health care providers was identified to increase community awareness and support advocacy for patients.

## Teaser

Survivors and community members in Hawassa, Ethiopia, gather for the first time to discuss breast cancer and advocacy.

## INTRODUCTION

Breast cancer (BC) is the most frequently diagnosed malignancy worldwide with the highest mortality in sub-Saharan Africa.^1-3^ In Ethiopia, most patients were age 35-45 years with late-stage disease, which is associated with suffering and premature death.^4,5^

**CONTEXT**

**Key Objective**
Breast cancer (BC) advocacy has been shown to strengthen policies and cancer screening, treatment, and research. How can BC advocacy be developed in the context of a city in southern Ethiopia?
**Knowledge Generated**
Survivors and influential community members in Hawassa City were assembled for the first time to explore challenges in BC and identify opportunities for advocacy work. Participants prioritized areas of needed work, explored trust-based relationships, and identified opportunities to develop strategic partnerships between religious leaders and health care providers.
**Relevance**
An ecological model was developed to organize findings at the individual, community, health system, and policy level. This approach of a community-based qualitative study in Hawassa, Ethiopia, could be used in similar settings wanting to develop BC advocacy.

Advocacy groups are critical for identifying unmet needs, challenges, and opportunities to achieve high-quality, patient-centered cancer care. They report disparities in access, quality, and outcomes. They also explore potential policy strategies to address barriers preventing equitable access to high-quality cancer care. Advocates help advance BC control and provide leadership in establishing BC priorities without government financial dependence.^6-8^

Advocates may be survivors, health care providers, cancer societies, and nongovernmental or civil society organizations who can be involved in decisions regarding BC care. Trust-based relationships and collaborations are the cornerstone of effective advocacy.^6^ Recommendations to improve BC advocacy emphasize knowledge, networking, strategic collaboration, training, organizational development, monitoring, and evaluation.^6,9-11^

Although the burden of BC is high in Ethiopia, there remain limited advocacy activities focused on BC in Hawassa City. Therefore, this study aimed to explore current BC challenges and identify opportunities for advocacy.

## METHODS

This study was conducted in Hawassa City, Ethiopia, between June and July 2022. Hawassa is located 273 km south of the capital city (Addis Ababa) and comprises 32 kebeles, eight subcities, and population of 394,057. There are five predominant ethnic groups: Sidama, Amhara, Welayta, Oromo, and Gurage. Approximately half of people speak Sidama as the first language and one-third speak Amharic. Christianity is the most common religion, followed by Islam.^12^

The Hawassa University Comprehensive Specialized Hospital Cancer Treatment Center (HUCSH-CTC) is the only regional comprehensive cancer center serving 25 million people from Sidama, Oromia, South, and Somale regions. HUCSH is a teaching and tertiary hospital with 520 in-patient beds. In 2013, the Breast Cancer Unit was established with six in-patient beds and staffed by two physicians and two nurses who completed short-term training in oncology. In 2016, fellows from the oncology training program in Addis Ababa traveled to Hawassa to provide care. In 2018, a graduate began providing care full time. In 2022, a new building (CTC) was constructed with 40 in-patient beds. In 2021, manual immunohistochemistry was launched at HUCSH, and the linear accelerator radiation machine is scheduled to become operational at the end of 2023. Currently the oncology unit has two medical oncologists, two oncology nurses, 20 nurses, a pharmacist, and holds BC tumor boards with international collaborators quarterly. Thus, capacity has increased over the past decade for comprehensive BC care to include surgery, chemotherapy, endocrine therapy, palliative care, immunohistochemistry, and soon radiation.

HUCSH-CTC has seen 100-120 BC cases per year, yet a recent study on regional care pathways showed that only 26% of patients with cancer were referred for treatment. Patients were 10 times more likely to complete referrals intended for diagnosis than those for treatment.^13^ Another study showed that the 3-year BC survival between 2013 and 2018 was 60%, with predictors of mortality including late-stage disease, rural residence, no receipt of surgery, and poor chemotherapy adherence. Most patients lived on farms and traveled 2-7 hours for treatment. Some patients also traveled to Addis Ababa for treatment after being on a waitlist over 1 year.^14^

We conducted in-depth interviews (IDIs) and focus group discussions (FGDs). Participants were selected on the basis of their potential to provide information related to BC challenges and opportunities for advocacy.^15^ All IDIs and FGDs were transcribed verbatim from the local language (Amharic) to English and codes were generated. The data analysis was managed using ATLAS.ti, version 7.5 (ATLAS.ti Scientific Software Development GmbH, Berlin, Germany).

Ethical approval was obtained from Hawassa University College of Medicine and Health Sciences institutional review board. Oral consent was obtained from participants.

## RESULTS

### Characteristics of the Participants

Twenty-five participants were included (Table 1). Health extension workers (HEWs) work in the community and in households to implement the Ethiopian Health Extension Package for the prevention and early detection of noncommunicable diseases, including cancer.^16^ The Women's Development Group (WDG) comprises volunteer community health workers who promote health and disease prevention. WDG members work closely with HEWs on health-related issues, linking and extending services from health post to households.

**TABLE 1:**
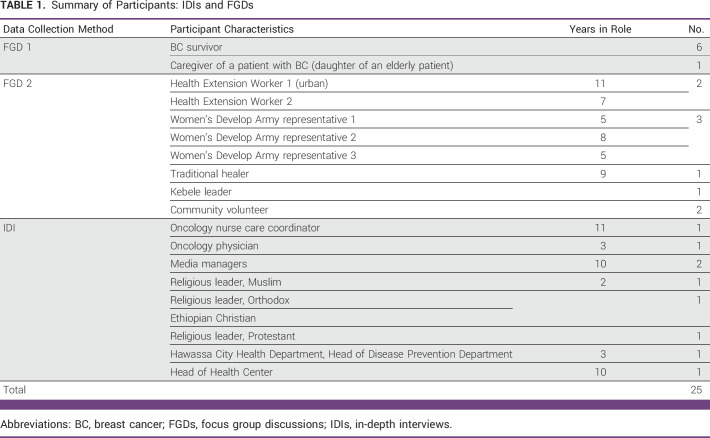
Summary of Participants: IDIs and FGDs

### Key Themes

Five themes emerged about BC challenges: inadequate knowledge in the community and stigmas about treatments such as mastectomy; insufficient media dissemination of information; community preferences for traditional medicine and religious practice over standard medical care; lack of funding and political commitment to prioritize BC; and patient challenges with costs of treatment, transportation, and social isolation. Key themes emerged about opportunities to begin advocacy work: community education should be promoted and patients, providers, and religious leaders were key sources of information, positive messages, and leadership. Trust-based relationships and opportunities for training, networking, and strategic collaboration were identified.

### Challenges

#### Inadequate Knowledge About BC in the Community

Most participants emphasized that although BC is a major public health problem, knowledge of the disease and treatments is low. Many participants stated that BC is perceived as a killer disease, curse from God, or death sentence.

*People are not willing to be screened. They fear it. There is a traditional meaning for cancer as ‘a curse.’…You can remember at the time HIV was symbolized as horror. Due to that, a lot of victims died and hid…All of my neighbors and friends go rid of me. There was a time when they refused to eat the food I prepared. They consider cancer contagious. (FGD, BC survivor)*

*[There is] confusion about the information from their family members and neighbors…The cancer patient shouldn't expose herself to family or friends during the initiation phase. They hurt her morale. (FGD, BC survivor)*

#### Stigmas About Treatments

Participants commented that mastectomy is feared and thought as ineffective.

*The majority of people, including the educated, don't allow breast surgery…I lost my sister due to her resistance to getting surgery. She had hidden it until cancer came out of her body…She was not able to show her body due to religious factors. (FDG, BC survivor)*

*The rural community and few of the educated argued with me for taking my mother to a health facility by saying, ‘How on earth do you allow breast surgery?’ (FGD, caregiver)*

*Women do not go to any health facility but if they go their breast could be cut without any good prognosis. (FGD, HEW)*

#### Preferences for Traditional Medicine and Religious Practices Over Modern Treatment

Participants commented on the connection between delays in care with seeking alternative therapies.

*They first select traditional medicine because it is cost-effective and can cure without cutting their breast…Because there is no medication for it rather than cutting breasts but in traditional medicine there is drinkable drugs and other drugs that can cure the cancer without cutting and applying any invasive procedure. (FGD, WDG)*

*I don't know how it was heard, people started to tell me to use holy water, traditional medicine, or stop the modern treatment. I was confused in the middle. I started to switch off my phone. (FGD, BC survivor)*

*Culturally, there are individual barriers. Our sisters and mothers are not willing to show their private areas where cancers are found…because of cultural and religious reasons. (IDI, Head, Health Center)*

#### Low Media Coverage of BC

The language about BC included descriptions of patients as victims who were attacked by cancer or a terrorist attack.

*Breast cancer is killing many mothers and sisters. It's a big public problem and community awareness and attitude is very poor. Our community still doesn't understand the disease so well. Much work is needed like COVID-19. Everybody must give attention to cancer because it can destroy human beings. (IDI, Media Manager)*

*Cancer is misled by the authorities. They give the name to the thieves…Those who are speaking about this on the media are shameless and uneducated. I had been living with cancer for 6 years now. I am almost recovered now. Had it been according to their words, I would have died by now. I suggest those who are speaking on the media calm down. It is very terrible. They should not use the cancer name for the terrorists… (FGD, BC survivor)*

#### Lack of Funding and Political Commitment to Prioritize BC

Many participants expressed the need for health policy and government guidance to address BC.

*Above all government gives attention to cervical cancer not for breast cancer…There are health professionals who have general knowledge and information but not details of breast cancer…[There is] limitation and inadequacy of service given at centers. Even those people who have been screened in different institutions do not know exactly where to go. There should be a good linkage system… (IDI, Head, Health Center)*

*The issue needs attention at country level not only at this town…There is only gossip about it in our town as well as a country level. We should give attention in our town as there are cancer patients and people dying from cancer…On this issue government must set a strategy on screening, treatment regarding breast cancer. We have no specific cancer budget and strategy regarding breast cancer. (IDI, Hawassa City Health Department)*

#### Patient Challenges With Financial Toxicity, Transportation, and Social Isolation

Survivors shared painful challenges.

*I am afraid when the people talk about the disease, Others say, ‘For God's sake don't cut your breast. It kills you whatever the treatment because it disseminates into the body.’ That kind of rumor caused mental stress on me. I had to hide from people because I'm afraid if they start talking about it…*

*It is only by the help of God, otherwise, according to their words that I would have not recovered. I was terrified a lot of the time…It took me a long time to get treatment; due to mental readiness. It was hard to initiate the treatment…Thanks to God, after all the challenges that I am here safely. The health insurance service is not always functional…A lot of patients die due to lack of medicine. Even gloves are purchased from outside. The suffering due to the lack of medicine is almost equal to the pain from the disease.*

*They rejected me in the end…Their father used to tell them I was valueless…My neighbors also advised my husband to divorce me.*

### Opportunities for Advocacy

#### BC Survivors as Sources of Information

This was the first time survivors had ever assembled and shared how they can promote health seeking behaviors to other women. Survivors expressed a desire for the community, health care facilities, and others to mobilize support for patients; however, the readiness for the survivors to actively participate or take on leadership roles in such activities was mixed. No survivor described having received support from nongovernment organizations or advocacy groups.

*If there is one who is attacked by cancer, she could have talked about it and become an initiation for others to be screened. That is how the information is disseminated gradually.*

*At the time people come to visit patients, they start to see themselves and say I have the same on my body too. Then we recommend they be screened.*

*The informal conversation from the so-called patient cannot be significant. Rather the advice from health professionals can be the source of information.*

*There are a lot of obstacles hindering patients from a health facility. One is economy, distance, and family support. I'd rather recommend them to form an association and seek treatment.*

*I am happy to get this interview. A lot has to be done about campaigning for cancer at the government level…You had better create a supporting system for those who are supported less. They should be supported by food. Medicine is costly. I know one woman who was thrown out of her rental house because she was not able to pay. Hence cancer should get attention and a campaign should be conducted.*

*I suggest on awareness creation…Husbands should take the responsibility of pushing their wives for screening. The shortage of medicine needs to be solved. Medicine should be available maybe with the help of the health bureau. I suggested at least an annual check-up that can hasten the early identification and treatment. They can use women who come for vaccinating their children.*

#### Increasing Community Awareness

Participants noted that media coverage should be inclusive of the diverse languages and cultures and use real scenarios and community examples.

*We have a large number of audience and also we have…websites, newspapers, TV/radio, and productions to achieve our audiences' demands. Currently we have 48 community languages…in our radio program. All of them have specific health problems including breast cancer. (IDI, media manager)*

*Educating the community by using banner**s** or leaflet**s** in their local language is very important and can support advocacy goals by working in collaboration with Women Development Team…Furthermore, providing health education using mass media can encourage women for self-examination and increases health seeking behavior if we teach the community about prognosis of breast cancer using real scenarios. (FGD, WDG)*

#### Credibility of Health Care Providers

Primary care providers were identified as trustworthy sources of health information, capable of networking across communities and the health care system, and advocating for patients.

*Including breast cancer as one of health extension workers packages, giving training for health professionals and working in collaboration with multiple organizations can have great effect on alleviating this problem. (FGD, WDG)*

*Lack of adherence for treatment is due to problems on referral linkage by HEW. We do have strong linkage and plan**s** for cervical cancer but we don't have for breast cancer…We can do home to home visit and we can educate women how to prevent and get service regarding breast cancer…We can teach and link them with health facilities. (FGD, HEW)*

*Even a wife cannot tell her cervical cancer problem to her husband. Rather she prefers to tell it to her similar sex…It is better to provide awareness at the health posts. (FGD, BC survivor)*

#### Religion and Medicine

Despite positive messaging from religious leaders there remained a preference for traditional and religious treatment over modern treatment. Participants identified the importance of discussing faith issues while raising community awareness about BC.

*This was a question that arose in connection with anti-HIV medication. A patient can pray without stopping the medication prescribed by the doctor. It's the same with cancer medicine…This is the teaching of the church…Obstacles can sometimes come from people in the leadership positions…If they don't understand the church teachings, they can say that if you only follow the spiritual you should leave the treatment. The second may originate from the believer. Because of lack of knowledge the believer may decide for himself that I believe in the creator and I should not do this. But it is possible to decrease it. It is necessary to go to the place where the problem is spread and works. If we properly educate the people, if we direct them, they will not leave us. (IDI, religious leader, Orthodox)*

*Some people say, ‘I don't need the treatment, I have a creator’ and then damage will come. Some of them have left their faith and are being treated. But it's better to run both connected and optimized. It is possible to pray while taking the medicine…I met someone here who has breast cancer. We sent her for treatment after convincing her that we will pray here and that she should get modern treatment…And the faith allows. If we work in harmony with other institutions, we can bring many changes. (IDI, religious leader, Muslim)*

#### Trust-Based Relationships and Strategic Collaboration

Opportunities were identified for religious leaders and health care professionals to collaborate in community outreach and support advocacy for patients.

*We (religious leaders) can give advice. Second there is something called reception. These people are between us and you (providers). We and you need to talk about this…It requires work from us and you. You must also teach. All religious institutions should teach it strongly because they are widely accepted by this society. Let’s all work together. (IDI, religious leader, Muslim)*

*Religious institutions are stakeholders…I see them (providers) discussing various diseases with governmental or non-governmental bodies. When there is a favorable situation in the church, I see them coming and providing awareness. (IDI, religious leader, Protestant)*

## DISCUSSION

To our knowledge, this is the first study exploring the context of BC in Ethiopia to understand possible advocacy approaches in a community with access to a tertiary comprehensive cancer center. Treatment cost, choice of traditional versus modern medicine, religious practices, and negative community attitudes (eg, stigma of treatment, social isolation) are major challenges for BC patients. Similar to previous reports in Ethiopia, hopelessness and uncertainty about the effectiveness of conventional medicine was common.^3,17-21^ These identified themes may influence the readiness for advocacy and advocacy-related activities in Hawassa among survivors and other community members. Notably, all participants acknowledged the great need to address BC challenges by raising community awareness, improving media coverage, supporting patients with BC, and strengthening health outreach at the primary care level.

Many respondents commented on the high financial burden of cancer treatments. This aligns with our previous study of patients with cancer in Hawassa (31% with BC) which found that the cost of cancer care was 56% of the average income. Costs included surgery, medication, and chemotherapy. Medications were the highest direct cost while transportation was the highest indirect cost.^22^

In this study, spirituality was a fundamental element of the healing process and comfort. Although patients and religious leaders supported both religious practice and medical care, they explained the community may perceive these as dichotomous options because of beliefs of BC as a curse; misunderstanding of religious teachings about medical care; or perceptions of medical care as unaffordable, unavailable, and ineffective to cure a curse.

Religious leaders, providers, and patients were identified as trustworthy sources of information who could change fatalistic perceptions of BC and the cycle of late-stage diagnosis leading to poor outcomes. Training and developing strategic partnerships between providers and spiritual leaders were identified as opportunities for community work. Spiritual care could be integrated into advocacy approaches and health care delivery from early detection to diagnosis, treatment, and long-term care.

**FIG 1:**
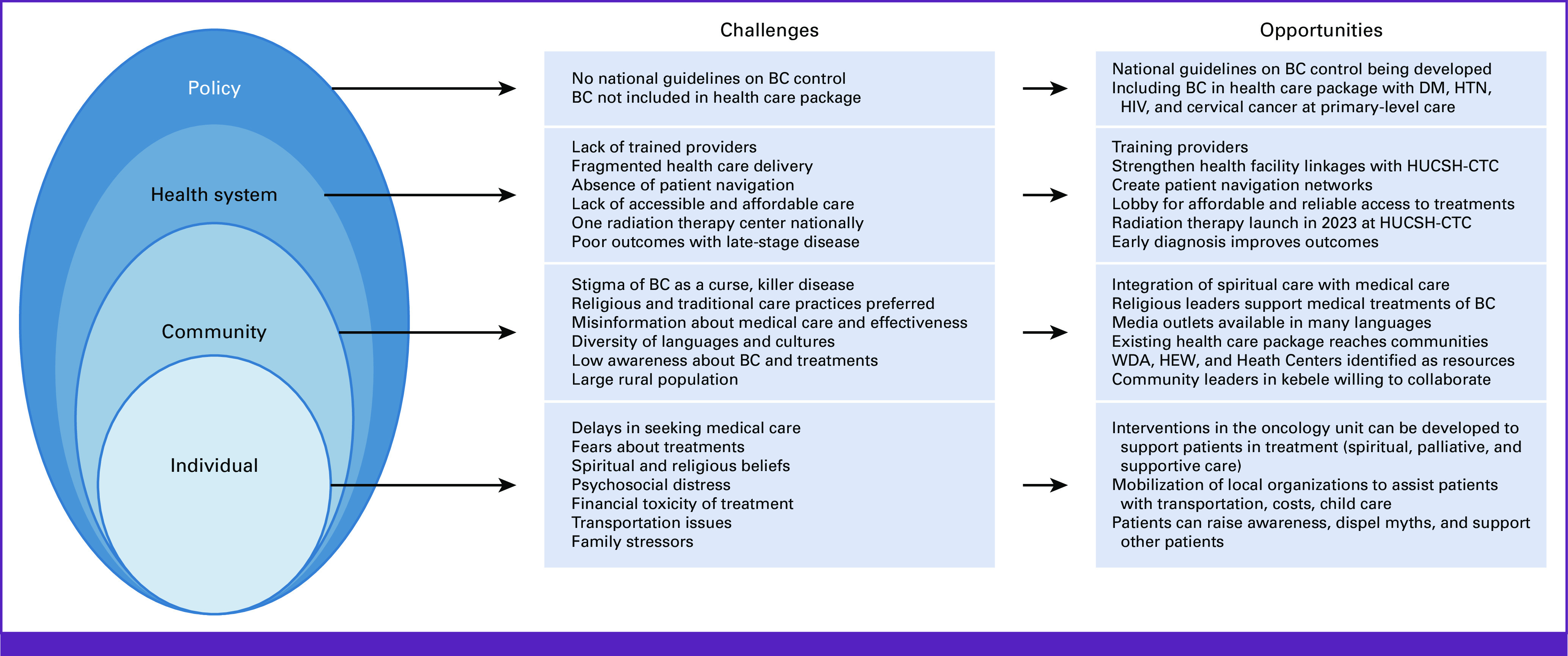
Ecological model of challenges and opportunities related to BC in Hawassa city, Ethiopia, to inform possible advocacy approaches. BC, breast cancer; DM, diabetes mellitus; HEW, health extension worker; HTN, hypertension; HUCSH-CTC, Hawassa University Comprehensive Specialized Hospital Cancer Treatment Center; WDA, Women's Development Army.

When BC is diagnosed at earlier stages, mortality is decreased.^23,24^ Implementing community-based interventions to increase early diagnosis and completion of treatments will require the inclusion of diverse perspectives such as those in this study. Figure 1 depicts an ecological model of behavior change, which can be used to inform future advocacy approaches in Hawassa and develop advocacy tools.^11^ Developing advocacy is especially timely because with launching radiotherapy, HUCSH-CTC will provide essential multimodality treatment, a major milestone that can improve BC outcomes.

Our study limitation was that it did not include the perspectives from advocacy groups or individuals self-identifying as advocates. In Hawassa City, there are limited active civil society organizations or nongovernmental organizations working to lobby or influence changes in the health sector. Those that do exist are not focused on BC specifically. The strength of this study was that it was the first time survivors and community members had ever assembled to discuss BC challenges as a starting point to develop the BC advocacy landscape in Hawassa.

In conclusion, recommendations for advocacy work in Hawassa include lobbying local policymakers to recognize BC as a health priority; including BC within the Health Extension Package of primary care programs; initiating programs to increase earlier detection and promote health care seeking behaviors; launching small group discussions in the community to change stigmas of BC and treatments; encouraging media to disseminate messages inclusive of people living in remote areas and speaking different languages; improving availability, affordability, and health care access; and providing psychosocial and spiritual support to women with BC. A strategic collaboration between religious leaders and health care providers was identified to increase community awareness and support advocacy for patients.
